# Constructing a Dual-Function Surface by Microcasting and Nanospraying for Efficient Drag Reduction and Potential Antifouling Capabilities

**DOI:** 10.3390/mi10070490

**Published:** 2019-07-23

**Authors:** Liguo Qin, Mahshid Hafezi, Hao Yang, Guangneng Dong, Yali Zhang

**Affiliations:** 1 Key Laboratory of Education Ministry for Modern Design & Rotary-Bearing System, Xi’an Jiaotong University, Xianning West Road, Xi’an 710049, China; 2 Institute of Design Science and Basic Component, Xi’an Jiaotong University, Xianning West Road, Xi’an 710049, China; 3 Key Laboratory of Biomedical Information Engineering of Ministry of Education, Xi’an Jiaotong University, Xianning West Road, Xi’an 710049, China

**Keywords:** shark skin, lotus-like structure, drag reduce, antifouling, hydrophobicity

## Abstract

To improve the drag-reducing and antifouling performance of marine equipment, it is indispensable to learn from structures and materials that are found in nature. This is due to their excellent properties, such as intelligence, microminiaturization, hierarchical assembly, and adaptability. Considerable interest has arisen in fabricating surfaces with various types of biomimetic structures, which exhibit promising and synergistic performances similar to living organisms. In this study, a dual bio-inspired shark-skin and lotus-structure (BSLS) surface was developed for fabrication on commercial polyurethane (PU) polymer. Firstly, the shark-skin pattern was transferred on the PU by microcasting. Secondly, hierarchical micro- and nanostructures were introduced by spraying mesoporous silica nanospheres (MSNs). The dual biomimetic substrates were characterized by scanning electron microscopy, water contact angle characterization, antifouling, self-cleaning, and water flow impacting experiments. The results revealed that the BSLS surface exhibited dual biomimetic features. The micro- and nano-lotus-like structures were localized on a replicated shark dermal denticle. A contact angle of 147° was observed on the dual-treated surface and the contact angle hysteresis was decreased by 20% compared with that of the nontreated surface. Fluid drag was determined with shear stress measurements and a drag reduction of 36.7% was found for the biomimetic surface. With continuous impacting of high-speed water for up to 10 h, the biomimetic surface stayed superhydrophobic. Material properties such as inhibition of protein adsorption, mechanical robustness, and self-cleaning performances were evaluated, and the data indicated these behaviors were significantly improved. The mechanisms of drag reduction and self-cleaning are discussed. Our results indicate that this method is a potential strategy for efficient drag reduction and antifouling capabilities.

## 1. Introduction

In marine infrastructures, such as ship hulls, wave-energy collectors, and undersea pipelines, high drag forces and biofouling are the two biggest pernicious effects [1,2,3,4,5]. Once fouling settlements are formed on immerged surfaces, they are very difficult to remove, even shortly after their formation. For a forward-moving watercraft, hydrodynamic drag and fuel consumption increase along with the occurrence of biofouling, which significantly decrease the operating speed and cruising distance. Previously, tributyltin self-polishing copolymer coatings were widely used to deal with biofouling on ships [6]. However, they were banned globally for the protection of the ocean environment and marine organisms due to their toxic properties. Thus, developing an effective and environmentally friendly antifouling system has been a subject of immense interest [7]. Many structures, materials, and surfaces observed in nature have inspired researchers to understand their basic principles, such as their intelligence, microminiaturization, hierarchical assembly, and adaptability. Some achievements, for example, rebuilding structures and materials of living creatures, have led to practical applications in the field of materials science and design [8,9]. 

Shark skin is a well-known example of a material that exhibits efficient drag reduction and antifouling performance. The skin contains minute individual features, called dermal denticles, which are parallel to the swimming direction. It has been proved that these microstructures reduce the formation of vortices, which results in water flowing easily across the shark skin [10]. The water layer near the skin moves faster and reduces the settling time of microorganisms. This eliminates the duration of fouling agglomeration and thus avoids the occurrence of strong adhesion between the substrate and fouling settlements. However, a certain number of microorganisms can still be found on shark skin. Some fouling settlements tend to adhere to particular grooves of shark skin, which is especially serious for artificially replicated shark surfaces. In nature, another well-known example of an antifouling material is the superhydrophobic lotus leaf. Water droplets from rainfall collect contaminated particles and carry them away to protect the leaves from pathogens [11,12]. Wax and hierarchical structures on the lotus leaf are reported to do this. Inspired by lotus leaves, numerous artificial superhydrophobic surfaces have been fabricated on various substrates such as tiles, textile, and glass [13,14]. Further, it was found that lotus-like materials and surfaces also reduce drag. To achieve superhydrophobicity, rough structures, especially the nanomorphologies, play a critical role. Silica is a widely researched nanoparticle which allows the formation of stable nanostructures. By spraying polymethyl methacrylate and a hydrophobic nanoscale silica compound onto a hydrophilic steel substrate, Wang et al. reported that effective drag-reduction behavior was found during a sailing test [15]. Tuo et al. showed that drag reduction was decreased by 20% on a superhydrophobic surface when the flow velocity was between 2 and 5 m/s [16]. However, compared with biomimetic shark-skin surfaces, the drag reduction of artificial lotus-like surfaces was much lower, and the mechanism of this effect is still under investigation. 

Recently, different technologies that introduce bio-inspired functions have been investigated to create surfaces that exhibit excellent and synergistic performances similar to living organisms. Additive manufacturing was used to design microriblet features, and a 3D-printed shark skin demonstrated almost 10% average fluid drag reduction [17]. Lu used chemical deposition to fabricate a micro–nanohierarchical structure for superhydrophobicity and drag reduction. The results indicated that the nanostructure may help the superhydrophobic surface exhibit drag reduction properties [18]. However, the connection between microscale and nanoscale features (such as the surface functions of shark skin and lotus-like structures) has not been completely revealed [12,19,20]. It is believed that superimposing patterns of a shark-skin surface (BS) and a lotus-like structure (LS) at the microscale and nanoscale, respectively, would produce a dual-function surface possessing both efficient drag reduction and antibiofouling performance. Therefore, the aim of this study was to develop a bio-inspired shark-skin and lotus-structure (BSLS) surface and for use in marine equipment applications. To achieve this, the hierarchical structure was introduced on polyurethane (PU) polymer. A vacuum casting procedure was selected to produce shark-skin-like micromorphologies. Mesoporous silica particles of a size similar to lotus-like structures were deposited on the replicated shark surface throughout the spraying process. The biomimetic surfaces were evaluated with various measurements. The morphologies were observed by scanning electron microscopy. The surface wettability was conducted by water contact angle (CA) measurements. Finally, antifouling, self-cleaning, and liquid drop impact experiments were carried out to illustrate the mechanism of their synergistic effect.

## 2. Materials and Methods 

The tiger shark (Heterodontus japonicus) was chosen for the template and was obtained from a fisherman in Xiamen, China. Shark skin from the posterior part was selected. 3-(aminopropyl) triethoxysilane (APTES) was used to disperse the nanoparticles and was purchased from Shanghai Macklin Biochemical Co., Ltd (Shanghai, China). Liquid polyurethane (PU, Sigma-Aldrich, Shanghai, China) was selected to replicate the shark skin. It was constituted with a two-component precursor and curing agent. Mesoporous silica nanospheres (MSN, diameter = 185 ± 30 nm) were purchased from Rizhao biotech company. The glutaraldehyde, alcohol, and acetone reagents were all analytical grade.

BS samples were fabricated by a typical vacuum casting process. Prior to the casting process, pretreatment of the shark skin was conducted. The soft tissue under the rigid shark skin was carefully removed. After the skin template was flattened and fixed to a rigid mold, it was immersed in 5% glutaraldehyde for over 2 h. Then, the template was rinsed with deionized water at least three times. Graded ethanol was used to conduct the dehydration process, which avoided deformation from excessive water loss during the typical drying process. Repeated rinsing was performed and concentrations of 30%, 50%, 75%, 80%, 95%, and 100% ethanol solution were applied every 20 min for each step. After keeping the template in a thermostat at 48 °C (Kewei 101, Beijing, China) for over 12 h, the pretreated template was ready for replication. 

**Figure 1:**
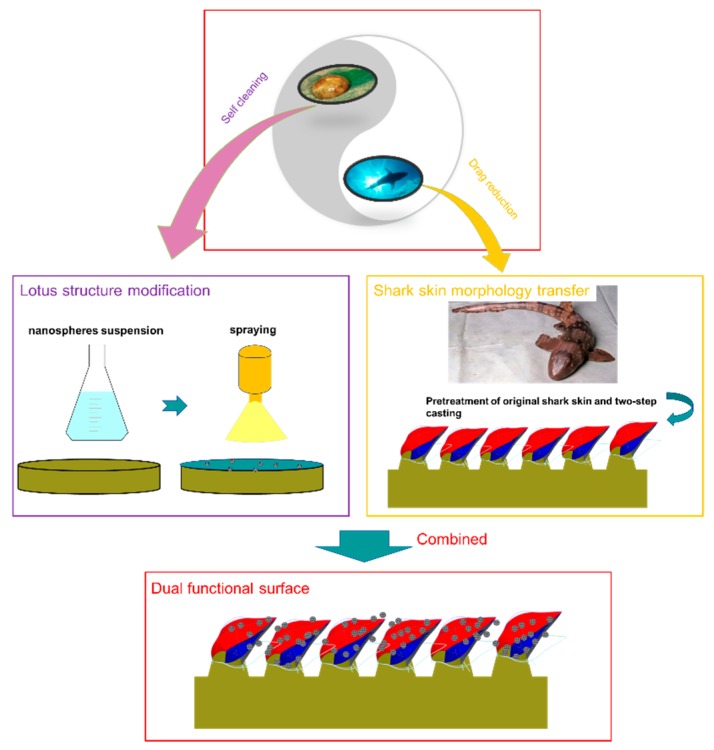
Process of synthetic replication of shark skin and lotus surface.

The detailed procedure for fabricating the BSLS surface is illustrated in Figure 1. Firstly, the treated template was localized on a plastic plate. A compound of liquid silicon precursor and curing agent was poured and the average of thickness of the liquid silicon was controlled to 2.5 mm. In order to make sure the liquid silica was fully adapted to the shark-skin template, all processes were conducted in a vacuum chamber. By carefully stripping the template, the silicon negative mold was obtained. Liquid polyurethane was poured and cured to complete the precise positive duplication. The third step was to spray the modified silica particles onto the replicated shark-skin surfaces to introduce the lotus structure. For the modification, mesoporous silica nanospheres were first dispersed uniformly in acetone solution. APTES with a concentration of 5% was added and used to modify the MSN. After sonication for 5 min, a good dispersion of MSNs was achieved and the radical groups of APTES strongly bonded the nanoparticles to the substrates [21]. The dispersed nanoparticles were sprayed on the BS surface by a spraying gun. Different concentrations were used to achieve various coverages of 3.75, 4.25, 5, and 7.5 × 10^−3^ mg/mm^2^. Samples were denoted as BSLS1–BSLS4, respectively.

The morphologies of the real shark skin, the negative mold, and the replicated PU samples were observed by field emission scanning electron microscopy (SEM, MAIA3 LMH, TESCAN, Warrendale, PA, USA) and 3D laser confocal microscopy (OLS4000, Olympus, Tokyo, Japan). The well-dispersed mesoporous silica nanospheres were checked by transmission electron microscopy (TEM, JEOL JEM-2100Plus, Tokyo, Japan). The chemical composition of the silica nanospheres was obtained with an energy-dispersive spectrometer (EDS, OXFORD instruments, Abingdon, UK) and attenuated total reflectance–Fourier transform infrared spectroscopy (ATR-FTIR) (TENSOR27, Bruker, Karlsruhe, Germany). 

Static CA was measured by a video-based contact angle system (JC2000D2A, Powereach, Shanghai, China) at room temperature. Young–Laplace fitting was applied to calculate the CA value. Three different regions were tested, and mean CA was regarded as the apparent CA. The effect of the proposed BSLS approach on the dynamic surface wettability was analyzed. The advancing contact angle and receding contact angle were calculated to obtain the contact angle hysteresis (CAH). For each CAH measurement, three different positions for each sample were tested.

**Figure 2:**
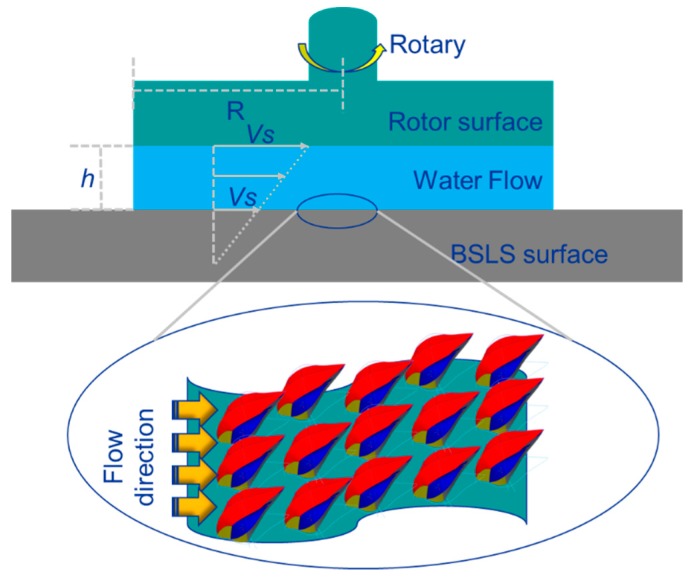
The schematic of the rheometer apparatus for measuring drag reduction, for which dermal denticles were oriented along the water flow.

The drag reduction of the BSLS surface was evaluated by a rheometer apparatus as described in [22,23]. Figure 2 shows the overview of the rheometer apparatus (MCR302, Anton Paar, Graz, Austria). The sample was fixed underneath the rotary plate. To keep the denticles in the same direction relative to the flow, the samples was cut into small parts and then they were oriented in the circle direction in the rheological experiment. We then carefully used the glue to adhere each part. Artificial seawater (1 mL) was injected into the gap of the rotary plate and BSLS surface. Here, the gap (h) was set to 1 mm and the rotation direction was along with the shark denticles. The velocity of the upper rotor plate was set to 100 rpm, and the drag reduction ratio was measured using the following equation:

$$\mathrm{Drag}\;\mathrm{reduce}\;\%=\;\frac{\tau_{\mathrm{nonslip}-}\tau_{\mathrm{slip}}}{\tau_{\mathrm{nonslip}}}$$

where $\tau_{\mathrm{nonslip}}$ and $\tau_{\mathrm{slip}}$ represent the shear stresses at the wall when no-slip and slip boundary conditions were used, respectively.

Self-cleaning properties were investigated by contaminating the BSLS surface with simulated pollution. Hydrophilic silicon carbide (SiC, Sigma-Aldrich, Shanghai, China) particles were used as simulated pollution. The size of the SiC particles ranged from 10 to 15 μm. SiC particles were chosen because of their performance similarity to natural dirt (shape, size, and hydrophilicity). SiC particles (300 mg) were gently sprayed onto the samples. Samples were localized on a tilted plate (30°) and water droplets were dripped from a specified height (30 ± 2 mm). The dripping was controlled at a constant rate (total duration of 1 min using 5 mL of water). The self-cleaning efficiency was calculated according to the weight variation before and after dripping.

Antifouling properties were evaluated by protein adsorption and protozoan colonization. For protein adsorption, samples were cut into blocks with size of 72 mm^2^ (6 by 12 mm) and soaked in a Bovine Serum Albumin (BSA) solution (0.5 mg/mL and pH ~7.4) for 6 h at room temperature. Using the Bradford method [24], the residual of BSA within the solution was measured. By subtracting its initial concentration, the adsorption of BSA was calculated. An ultraviolet spectrophotometer (UV-3600, Shimadzu Co., Kyoto, Japan) with a wavelength of 595 nm was used to measure the absorbance of the solutions. The amount of adsorbed BSA protein on the samples was represented in the form of μg/cm^2^. For protozoan colonization, samples were cocultured with protozoan suspension with a concentration of 1 × 10^3^ protozoans/mL. After 48 h, samples were taken out, washed with distilled water, and observed under an optical microscope. The number of viable protozoans was counted and three different points were measured for each sample.

To evaluate the robustness of the biomimetic surface, samples were exposed to water with continuous impacting. The velocity was set to 1.0 m/s. After every hour, samples were taken out and dried totally. The contact angle was measured. For all the wettability tests, 5 μL of distilled water was dipped on the specimens by a microliter syringe, and each specimen was tested thrice to obtain the mean value. We further measured the mechanical robustness of the BSLS surface. Sandpaper abrasion was used to study the wear resistance properties. A 1 by 1 cm^2^ sample was fixed on the bottom of a 50 g weight (4.9 KPa). The sample was then uniformly slid onto 600 grit sandpaper (Zhangshi Co., Shenzhen, China). Each abrasion cycle spanned 10 cm in a forward motion. After 100 cycles, the worn morphologies were observed under SEM.

## 3. Results and Discussion

### 3.1. Surface Characterization

**Figure 3:**
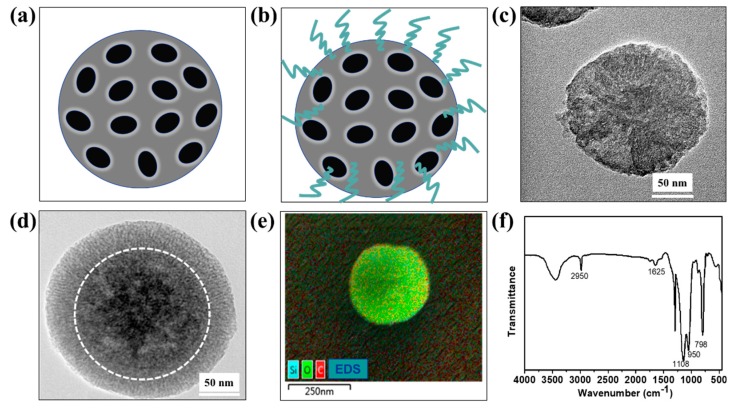
Mesoporous silica nanospheres used in the study. (**a**,**b**) are illustrations of bare mesoporous silica nanospheres and mesoporous silica nanospheres modified with 3-(aminopropyl) triethoxysilane (APTES). (**c**,**d**) are the corresponding transmission electron microscopy (TEM) images, (**e**) chemical composite, and (**f**) Fourier transform infrared spectroscopy (FTIR) spectrum of mesoporous silica nanospheres modified with APTES.

Mesoporous silica nanospheres were selected due to their morphological similarity to lotus-like structures and ease of chemical modification with biomolecules. Additionally, mesoporous silica nanospheres are theranostic agents and carriers for drug delivery [25,26], which could be considered as a potential application in loading antifouling agents for release. TEM was used to observe the fine features of the mesoporous silica nanospheres. As shown in Figure 3a,c, the diameter of the nanospheres was in the range of 162–185 nm and a hollow structure was observed. After modification with APTES, the diameter was in range of 228–261 nm (Figure 3b,d). The EDS map of one single nanosphere confirmed that it mainly consisted of Si and O elements (Figure 3e). Carbon elements were also observed, which indicated that APTES was successfully grafted on the silica nanosphere. The FTIR spectra of modified silica nanospheres are shown in Figure 3f. The typical absorption peaks of SiO_2_ materials were observed, such as Si–O–Si symmetric stretching (798 cm^−1^), Si–OH asymmetric vibration (950 cm^−1^), and Si–O asymmetric vibration (1108 cm^−1^) [27]. The peak at 2950 cm^−1^ was the C–H stretching, which further confirmed the grafting of APTES.

**Figure 4:**
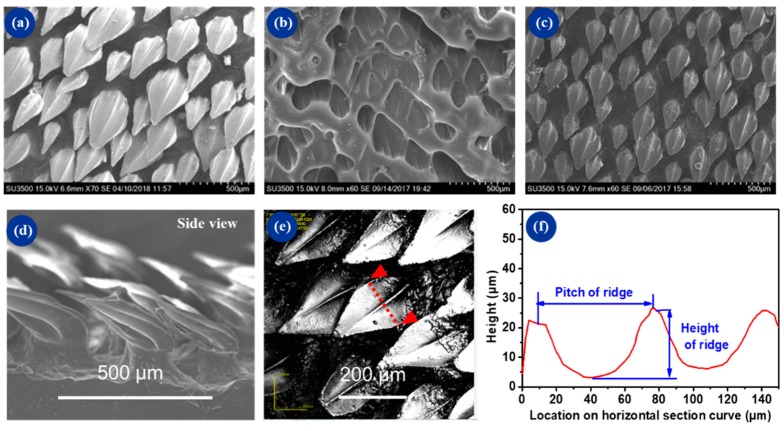
SEM and surface profile of shark skin, negative mold, and replicated polyurethane (PU) coating: (**a**) pretreated shark skin, (**b**) negative mold, (**c**) replicated shark surface, (**d**) side view of dermal denticles, (**e**) confocal microscope image, and (**f**) the section profile of dermal denticles that was marked in (**e**).

Figure 4 shows the morphologies of the original shark skin, the negative mold, and the replicated PU substrate. The replicated PU sample (Figure 4c) exhibited almost the same surface features as that of the denticles on the real shark. Figure 4b shows the negative mold of silicone, which showed more well-defined structures and higher integrity than the original shark skin. This was attributed to the good seepage characteristic of liquid silicone, which was capable of filling in the cavities of the mold before it was fully cured. As shown in Figure 4f, the horizontal curve section describes the central section of one single denticle (red line marked in Figure 4e). The pitch of the groove was around 70 μm and the height was around 25 μm. The results indicated the high replication precision of geometrical morphology, and the obliquity of the scales was maintained during the fabrication process.

**Figure 5:**
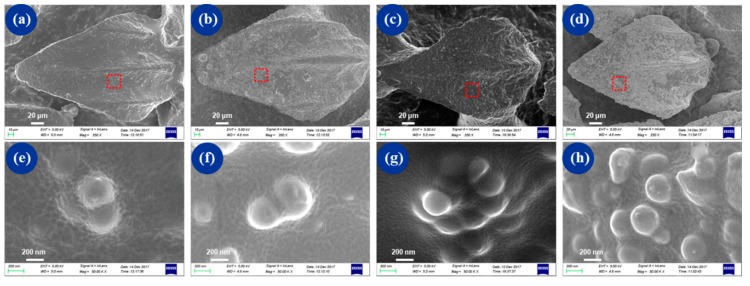
SEM micrographs were taken at a 10° tilt angle showing the morphologies of the shark-skin and lotus-structure (BSLS) samples with different concentration modifications of mesoporous silica nanospheres: (**a**–**d**) represent 3.75, 4.25, 5, and 7.5 × 10^−3^ mg/mm^2^, respectively; (**e**–**h**) correspond to the red area in (a–d).

Micrographs of nanospheres at different concentrations on dermal denticles are shown in Figure 5. A homogeneous distribution of mesoporous silica nanospheres was observed on all samples. As the spraying concentration of mesoporous silica nanospheres increased, more lotus-like structures were found on dermal denticles. The results imply that the BSLS surface possessing dual biomimetic morphologies was successfully fabricated. By combining microcasting and spraying, the BSLS surface exhibited the surface features of shark skin and hierarchical lotus-like structures. By adjusting the concentration of MSN, various coverages of lotus-like structures were achieved.

### 3.2. Surface Wettability

**Figure 6:**
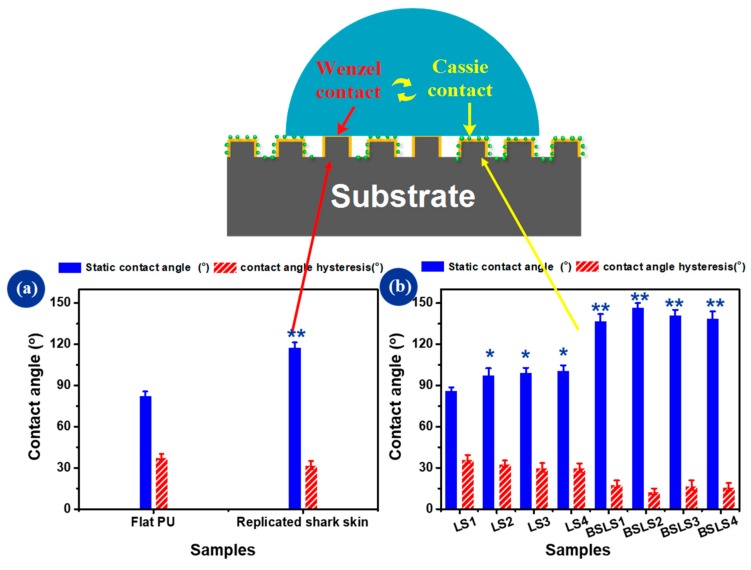
Bar charts showing the measured static contact angle and contact angle hysteresis. Four different spraying concentrations were used for the flat PU and replicated shark skin: 3.75, 4.25, 5, and 7.5 × 10^−3^ mg/mm^2^. Samples were denoted as lotus-like structure LS1–LS4 and BSLS1–BSLS4, respectively. (**a**) static contact angle and contact angle hysteresis for Flat PU and BS samples, (**b**) static contact angle and contact angle hysteresis after spraying SiO_2_ * *p* < 0.05 different and ** *p* < 0.01 significantly different from contact angle (CA) measured on flat PU by One-way ANOVA.

To investigate the effect of mesoporous silica nanospheres on the surface wettability, the static contact angle, and contact angle hysteresis were measured on the BSLS samples. As shown in Figure 6a, flat PU had a contact angle around 83.1°, indicating hydrophilicity. The contact angle of the replicated shark-skin surface was 118.1° and increased by 42% compared with that of flat PU. The contact angle hysteresis for flat PU and replicated shark skin was higher than 30°, which indicated that there was high adhesion between the surface and water. After being sprayed with nanospheres, the contact angles for all substrates were increased. When the flat surface was modified with MSNs, the surface wettability improved slightly, for example, 86.6° for LS1 and 98.2° for LS2. For the replicated shark skin, the contact angle increased dramatically to 147.2° with the MSN modification (e.g., the BSLS2 sample) and the surface was close to being superhydrophobic. Importantly, this combination has been shown to create a rough surface structure and low-surface-energy nanospheres when fabricating superhydrophobic surfaces. As it was observed, the dual-structured surface for the contact angle hysteresis had a lower value compared with that of the flat and replicated shark-skin surfaces, respectively.

To effectively fabricate a water-repelling surface, an effective air film between the surface and water droplets is fundamental. Here, air was easily trapped by the rough structure of the samples. The wetting mode of the hydrophilic surface could be considered using the Cassie–Baxter wetting mode, which is mainly used to describe a heterogeneous wetting regime [28]. In this study, the prepared BSLS surface was considered as a typical composite solid–air surface. Proof was provided by the Cassie–Baxter equation [29,30]:

$${\cos\theta }_{w}=\Phi_{s}\left({\cos\theta }_{e}+1\right)–1$$

where Φ_s_ is the area ratio between liquid and solid contact. It built the relationship between the apparent contact angle (θ_w_) of the composite interface and its intrinsic contact angle (θ_e_). The surface became more hydrophobic as Φ_s_ increased. A large fraction of air could be trapped by interstices of the microstructures. Water droplets were mainly localized on peaks of the surface and had difficulty penetrating the interstices between the different topographical peaks. With the MSN modification, the synergistic effect of microroughness and MSNs may have caused the lower CAH. These results indicate that surface morphology, especially the nanoscale features, had a more significant effect on controlling the surface wettability. However, this enhancement was not obvious when the number of nanospheres was higher than 5 × 10^−3^ mg/mm^2^. This could have been due to fewer morphological changes if the concentration of nanospheres was increased to a certain value and a certain amount of aggregation. At the same time, the structures, such as those shown in Figure 5c,d, did not trap sufficient air in the structure for a low CAH and high CA.

### 3.3. Drag Reduction Performance

**Figure 7:**
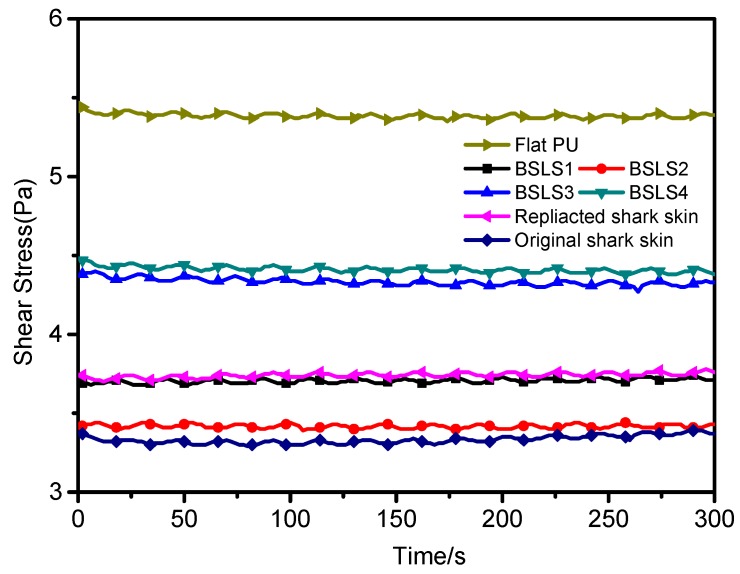
The shear stress for different samples.

The drag reduction behavior of the BSLS surface was characterized by a rheometer apparatus. Figure 7 shows the shear stress under constant flow velocity in the simulated seawater environment. In the test condition, the mean shear stress of flat PU was 5.41 Pa, which was the highest among all samples. For the replicated shark surface, the mean shear stress was 3.73 Pa. It decreased by 31%, exhibiting a good reduction of shear stress and drag force. When the replicated shark surface was further modified with MSNs, all surfaces showed lower shear stress values compared with that of flat PU. For a lower concentration modification, the shear stress was decreased (i.e., BSLS1 and BSLS2) even less than that of the replicated shark surface. The drag reduction of BSLS2 was lower than 36.7%, which was near that of the original shark skin (39.0%). When the concentration was further increased (i.e., BSLS3 and BSLS4), the shear stress decreased less. The drag reduction was 20.2% and 18.5% for BSLS3 and BSLS4, respectively. Generally, the relative velocity was considered to be zero at the boundary between the solid wall and liquid. This was widely characterized as a no-slip boundary condition. In our study, the velocity of fluid appeared to a nonzero for BSLS surfaces. An air layer was considered to form in interstices of the biomimetic surface. This layer acted as an air pad and a shear-reducing boundary was formed between the solid surface and the fluid, which significantly reduced the drag force. Previous works have shown that a shark-skin-like surface encourages the formation of secondary small eddies inside the riblet spacing [31,32,33]. A more detailed investigation of the effect of the BSLS surfaces on drag reduction is presently being carried out.

### 3.4. Antifouling and Self-Cleaning Performance

**Figure 8:**
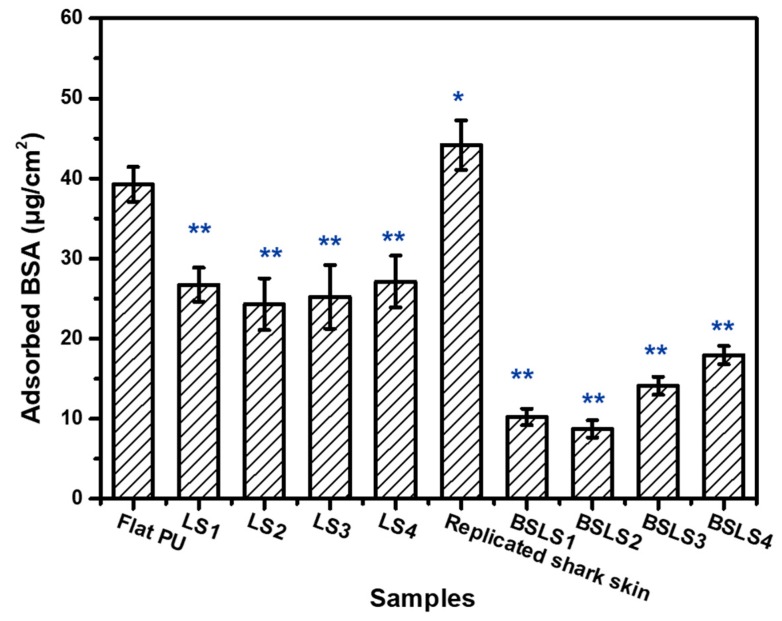
The amount of adsorbed BSA on the surface measured by the Braford method. * *p* < 0.05 different and ** *p* < 0.01 significantly different from adsorbed BSA measured on flat PU by One-way ANOVA.

Protein adsorption is the first step of biofouling, which is undesirable for most marine applications. Evaluating the adsorption amount of protein on a surface is regarded as a common way of estimating antibiofouling performance. For the antifouling study, the BSA adsorption of flat PU, replicated shark skin, SiO_2_-nanosphere-modified PU, and BSLS samples was measured. As shown in Figure 8, the BSA adsorption varied for the different samples. For the replicated shark-skin surface, the microroughness increased the BSA adsorption (44.2 μg/cm^2^) due to the enhanced surface contact area. The microroughness provided more sites for the adsorption of protein molecules [22]. With SiO_2_ nanosphere modification, the amount of BSA adsorption decreased. The lowest adsorption level was observed on BSLS2 (8.75 versus 39.27 μg/cm^2^ for flat PU), indicating a significant enhancement in resistance to protein adhesion. This can be attributed to the enhanced hydrophobicity due to the combination of replicated shark skin and SiO_2_ nanospheres. As a result of nanoscale roughness (SiO_2_ nanospheres), protein resistance was expected to be increased due to the water barrier, which would be caused by the enhancement of superhydrophobicity [34,35]. A physical barrier would form to prevent direct interaction between the surface and the protein. The intermediate wettability of the nanostructured surfaces would promote and induce the conditions for forming protein aggregates and nucleation. In this study, the increase in nanoscale roughness alongside the enhancement of hydrophilicity was the main reason (e.g., decreasing ~78% of protein adsorption for biomimetic surfaces (BSLS2)). Additionally, the surface negative charge was improved due to the hydrolyzed hydroxyl groups, which were derived from the deposited SiO_2_ nanoparticles. These generated a synergistic improvement of protein resistance [36,37]. Meanwhile, the sliding time of droplet was measured when samples was fixed on the tiled platform. As shown in Movie S1 (Supplementary Materials. Movie S1), for the flat and LS samples, both of water and milk were stuck on the surface. On the contrary, less adhesion and short sliding time were observed on the replicated shark skin and BSLS surface. On a 12 mm sliding distance, it took 610 ms for the replicated shark skin while it took 360 ms for BSLS surface when the test medium was water. Similar phenomenon was observed when the test medium was milk.

Settled protozoans were visualized by a Zeiss optical microscope. As shown in Figure S1 (Supplementary Materials. Figure S1), the highest density of settled protozoans was observed on the BS surface. A significantly lower density of protozoans was found on the LS surface. Compared with the flat sample, the settlement density was further reduced on the BSLS surface. Protozoa adhesion was a dynamic process and was influenced by various factors. The microstructure of BS provided a sufficient location for protozoa settlement. Protozoans accumulated on the BS surface. After SiO_2_ was deposited, the physical barrier and lower surface energy made the BSLS surface ideal for antifouling.

The self-cleaning performance of the samples was evaluated by measuring the residual mass of the pollutants after washing. The reduced residual amount was due to the better antifouling performance of the samples. As shown in Table 1, the residual mass of pollutants for the modified surface decreased compared with that of the flat surface. The residual mass of pollutants was 13.4 mg for the flat surface. Due to MSN spraying, the residual mass of the contaminant was significantly decreased, almost less than 30% of the flat sample. For the BS surface, the residual mass was slightly reduced by about 23%. However, for the dual biomimetic BSLS surface (e.g., BSLS2), the pollutants were almost removed.

### 3.5. Surface Duration

**Figure 9:**
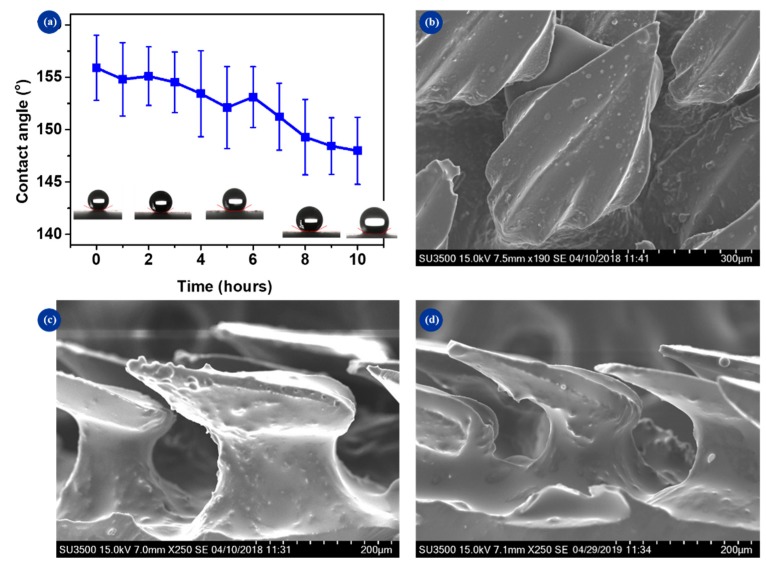
Durability of the biomimetic surface. (**a**) The evolution of contact angle with water impacting, (**b**,**c**) top and section views of the surface after 4 hours’ impacting, and (**d**) section view of the surface after 10 hours’ impacting. The sample of BSLS2 was used.

Considering most practical applications, functionality and superhydrophobicity must remain stable for a long time, which is very difficult to achieve. To evaluate their robustness, the samples were exposed to a sustained water flow. The speed was controlled at 1.0 m/s and the duration of impacting was 10 h. The evolution of the contact angle is shown in Figure 9a. It can be observed that the surface wettability of BSLS was quite stable. After 10 h of water flow, the contact angle slightly decreased from 156° to 145°. Commonly, the hydrophobic property is gradually weakened after exposure to humid conditions for a few hours. Also, if MSNs were lost or removed by water impacting, it would influence the performance of the BSLS surface (e.g., a sharp decrease of the water contact angle). The results showed that the finished surface was almost the same hydrophobicity of the initial surface. There was little damage to the nanomorphologies after a long duration of flow impacting, which indicated that the BSLS surface exhibited long-term stabilization. The integrity of the exposed surface was further verified by SEM, as shown in Figure 9b,c. The top and section views confirmed that the biomimetic surface retained a similar profile to when it was prepared. One reason for this was the strong interaction between SiO_2_ nanospheres and the replicated shark skin. Another reason was the relevant reduction of flow drag caused by the effect of the dermal denticles. An interesting observation was that when the water jet impacted the samples, it rapidly bounced off their surface. This phenomenon indicated that the typical Cassie–Baxter wetting state existed on the biomimetic surfaces, which extremely weakened the interaction between the BSLS surface and water. Thus, less damage occurred.

**Figure 10:**
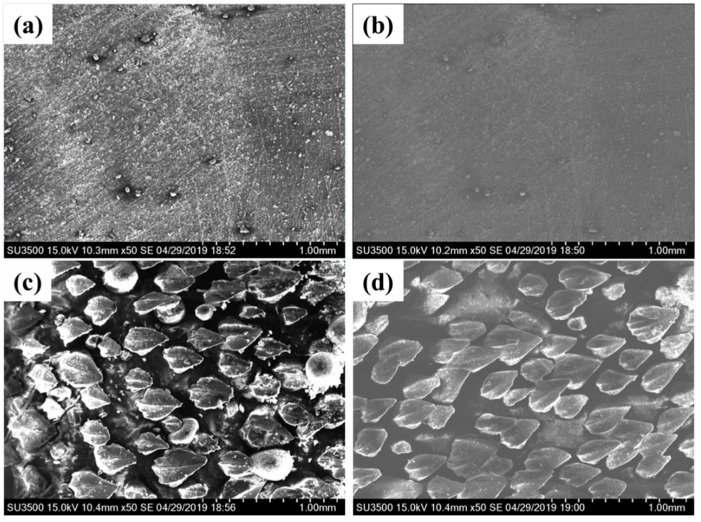
SEM of surfaces after abrasion with sand paper: (**a**) flat sample, (**b**) LS sample, (**c**) BS sample, and (**d**) BSLS sample.

We further measured the mechanical robustness of the BSLS surface. As shown in Figure 10, the flat PU was covered with various ploughs. With MSN modification, the wear resistance was significantly improved and less ploughs were observed. For the BS surface, several riblets were severely damaged, while the surface remained stable for the BSLS sample (Figure 10d). Compared with the flat surface, the wear resistance of the BSLS surface was greatly improved. 

### 3.6. Antidrag and Antifouling Mechanism

**Figure 11:**
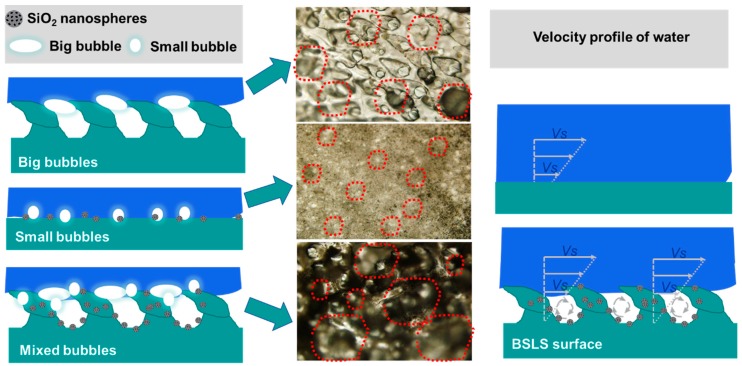
Antidrag and antifouling mechanism.

The experimental evidence showed that the BSLS surface with an optimized nanosphere concentration achieved significant drag-reduction and fouling-resistance effects, thus demonstrating its potential application in decreasing energy consumption during sailing. Many studies have confirmed that surface wettability is regulated by surface roughness [30,38]. In this study, the biomimetic morphology was fabricated on PU. Superhydrophobicity was achieved by the synergistic effect of the replicated shark skin and MSNs. Previous researchers have revealed that drag is mainly influenced by the slippage between the liquid and the contacted solid [23,39]. For the hydrophobic surface, air layers existed according to the Cassie model. When the biomimetic sample was immersed in distilled water, the surface was wrapped by different types of bubbles, as shown in Figure 11. As marked in the red area, small bubbles were mainly observed on the LS surface, while big bubbles were mainly observed on the replicated shark-skin surface. For the BSLS samples, both small and big bubbles were observed. The slippage of the specimen underwater was the result of interactions among the molecules of the solid surface, air bubbles, and liquid, which were regulated by the surface roughness, surface wettability, and shearing rate. Commonly, air bubbles between the solid and liquid interface is the main reason for increasing the slip length. 

Combining the results of the water impacting experiments and the contact angle measurements, the slipping of water indicated that the chance of forming large air bubbles was improved on the replicated surface, especially for the BSLS surface. This enhanced effect makes the liquid and even contaminants more easily removed from the top of the contacted surfaces. As described in our and others’ research, kinematic viscosity notably declined and, thus, self-cleaning was achieved, which converted the solid–liquid contact mode into the solid–air–liquid mode [40]. Moreover, small bubbles merge in bigger bubbles or thick air layers, which acted as a barrier or air pillow and caused adhesion to lessen [26,41].

## 4. Conclusions

To create durable bio-inspired surfaces with superhydrophobic, antidrag, and self-cleaning properties, dual biomimetic morphologies were developed for use on commercial PU. Two steps were involved in the fabricating process: Microcasting and nanosphere spraying, which created both the shark-skin microdenticles and the lotus-like nanofeatures. The results showed that it was possible to realize a larger area and high replication precision by microcasting. The subsequent spraying made the samples exhibit hybrid micro/nanostructures with excellent replication of the shark-skin microstructure. The spraying amount was the key factor affecting and controlling the CA and CAHs. At the concentration of 4 × 10^−3^ mg/mm^2^, the dual biomimetic surface showed near superhydrophobicity and the lowest contact angle hysteresis. The water contact angle was 147°, which was 29° higher than that of the unsprayed BS surface. The BSLS2 specimen exhibited excellent drag-reduction and self-cleaning performances. Compared with flat PU, the drag reduction and self-cleaning for BSLS2 were almost increased by 36.7% and 76%, respectively. The robustness of the BSLS surface was verified by sandpaper abrasion and long-term water impacting. The air-bubble layer that existed at the contact surface was believed to have had a significant impact on the drag-reduction and self-cleaning performances. This surface manufacturing technique is fast and feasible and results in the surface possessing better self-cleaning, antifouling, and drag-reduction capabilities. It is believed that this method is an effective strategy that has promising industrial applications for practical utilizations.

## Figures and Tables

**Table 1:** Measured residual mass of the samples.

Samples	Flat PU	LS1	LS2	LS3	LS4	Replicated Shark Skin	BSLS1	BSLS2	BSLS3	BSLS4
Δm (mg)	13.4 ± 2.3	12.1 ± 2.4	12.5 ± 2.6	13.2 ± 3.1	13.1 ± 2.9	9.2 ± 1.9	3.5 ± 0.9	3.1 ± 0.8	3.4 ± 1.1	4.1 ± 1.2

## Supplementary Materials

The following are available online at https://www.mdpi.com/2072-666X/10/7/490/s1, Figure S1: Optical images of protozoa adhesion on different samples. (a) flat sample, (b) LS, (c) BS, and (d) BSLS. The scale bar is 300 μm. The red circle shows the location of protozoa. Video S1: Sliding tests of the dual bio-inspired shark-skin and lotus-structure surfaces.
