# Supplementary material for: Constructing a Dual-Function Surface by Microcasting and Nanospraying for Efficient Drag Reduction and Potential Antifouling Capabilities
Source: Micromachines (Basel). 2019 Jul 23;10(7):490. doi: 10.3390/mi10070490 (PMC6680531; doi:10.3390/mi10070490)
Supplement: Supplementary file 1 [file micromachines-10-00490-s001.zip › supplementary/micromachines-527981 - sup.docx]

For protozoan colonization, samples were cocultured with protozoan suspension with a concentration of 1 × 10^3^ protozoans/mL. After 48 h, samples were taken out, washed with distilled water, and observed under an optical microscope. The number of viable protozoans was counted and three different points were measured for each sample.

As shown in S1, the highest density of settled protozoans was observed on the BS surface. A significantly lower density of protozoans was found on the LS surface. Compared with the flat sample, the settlement density was further reduced on the BSLS surface. Protozoa adhesion was a dynamic process and was influenced by various factors. The microstructure of BS provided a sufficient location for protozoa settlement. Protozoans accumulated on the BS surface. After SiO_2_ was deposited, the physical barrier and lower surface energy made the BSLS surface ideal for antifouling.


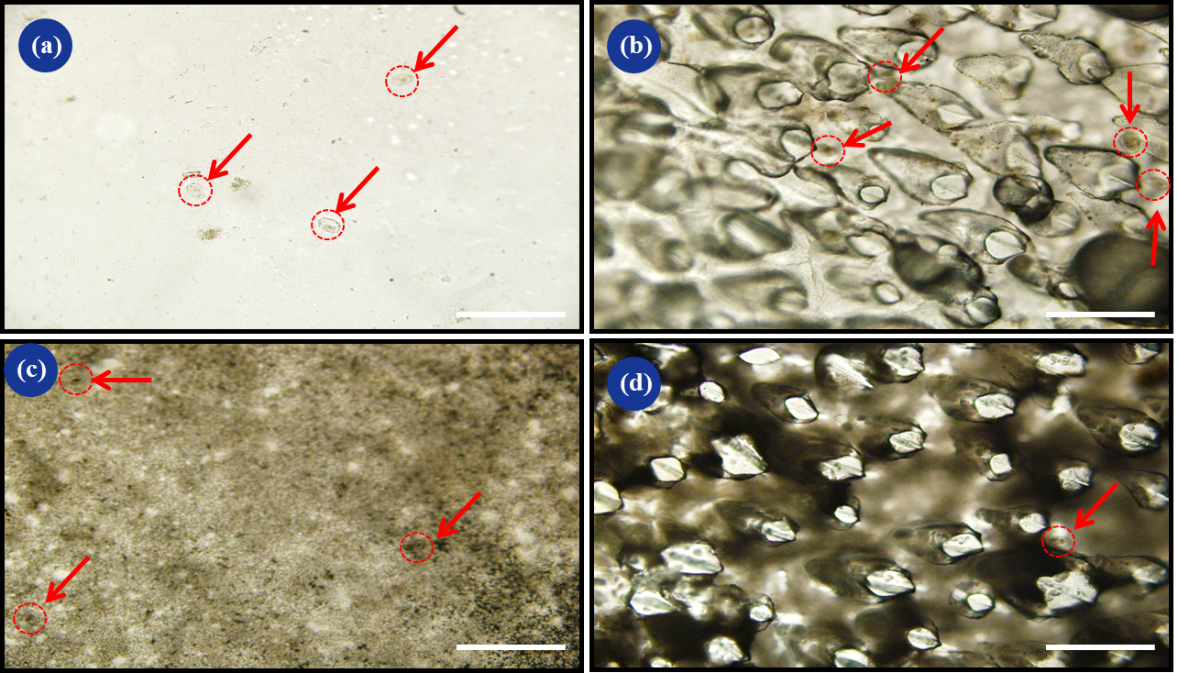


**Figure S1.** Optical images of protozoa adhesion on different samples. (**a**) flat sample, (**b**) LS, (**c**) BS, and (**d**) BSLS. The scale bar is 300 μm. The red circle shows the location of protozoa.
